# Integrating molecular, phenotypic and environmental data to elucidate patterns of crocodile hybridization in Belize

**DOI:** 10.1098/rsos.150409

**Published:** 2015-09-30

**Authors:** Evon R. Hekkala, Steven G. Platt, John B. Thorbjarnarson, Thomas R. Rainwater, Michael Tessler, Seth W. Cunningham, Christopher Twomey, George Amato

**Affiliations:** 1 Department Biological Sciences, Fordham University, New York, NY 10458, USA; 2 Sackler Institute for Comparative Genomics, American Museum of Natural History, New York, NY 10024, USA; 3 Richard Gilder Graduate School, American Museum of Natural History, New York, NY 10024, USA; 4 Wildlife Conservation Society, 2300 Southern Boulevard, Bronx, NY 10460, USA; 5 Baruch Institute of Coastal Ecology and Forest Science, Clemson University, PO Box 596, Georgetown, SC 29440, USA; 6 C2Me Engineering, 2744 Santa Claus Drive, South Lake Tahoe, CA 96150, USA

**Keywords:** American crocodile, Morelet’s crocodile, hybrid zone, hybridization, species' boundaries, Belize

## Abstract

The genus *Crocodylus* comprises 12 currently recognized species, many of which can be difficult to differentiate phenotypically. Interspecific hybridization among crocodiles is known to occur in captivity and has been documented between some species in the wild. The identification of hybrid individuals is of importance for management and monitoring of crocodilians, many of which are Convention on International Trade in Endangered Species (CITES) listed. In this study, both mitochondrial and nuclear DNA markers were evaluated for their use in confirming a suspected hybrid zone between American crocodile (*Crocodylus acutus*) and Morelet’s crocodile (*Crocodylus moreletii*) populations in southern Belize where individuals and nests exhibiting atypical phenotypic features had previously been observed. Patterns observed in both phenotypic and molecular data indicate possible behavioural and ecological characteristics associated with hybridization events. The results of the combined analyses found that the majority of suspected hybrid samples represent crosses between female *C. acutus* and male *C. moreletii*. Phenotypic data could statistically identify hybrids, although morphological overlap between hybrids and *C. moreletii* reduced reliability of identification based solely on field characters. Ecologically, *C. acutus* was exclusively found in saline waters, whereas hybrids and *C. moreletii* were largely absent in these conditions. A hypothesized correlation between unidirectional hybridization and destruction of *C. acutus* breeding habitats warrants additional research.

## 1. Introduction

Crocodiles (*Crocodylus* spp.) hybridize readily in captivity [1–5], and hybridization is known or suspected to occur among wild populations of several sympatric species [6–12]. Despite hybridization being considered a potential threat to some populations of endangered crocodilians [3,4,9,10,12–14], the frequency, geographical extent and drivers of hybridization among wild crocodilians remain poorly understood [11,15].

Hybridization between the American (*Crocodylus acutus*) and Morelet’s (*Crocodylus moreletii*) crocodiles was long postulated based on observations of crocodiles with phenotypic characteristics of both species [16–24]. More recently, molecular tools have provided genetic evidence for hybridization between these species in northern Belize [8] and the Yucatán Peninsula of Mexico [10,11]. In Mexico, hybridization appears to occur primarily in coastal regions of sympatry [10,11], while in Belize hybrids were found at inland sites outside the distribution of *C. acutus* [8]. Hybridization between *C. acutus* and the endangered Cuban crocodile (*Crocodylus rhombifer*) has been determined to be much more extensive than previously assumed based on phenotypic data [12].

Integrating molecular, phenotypic and environmental data to elucidate patterns of crocodile hybridization in Belize is important as the International Union for Conservation of Nature (IUCN) [25,26] currently classifies *C. acutus* as vulnerable (globally) and *C. moreletii* as conservation dependent. In this study, we use mitochondrial sequence and nuclear microsatellite data to assess the correspondence between phenotypic characteristics of nests, eggs and crocodiles with genetically based species assignment for populations of *C. acutus* and *C. moreletii* in coastal regions of Belize. We also examine ecological features associated with the presence of hybrid crocodiles and potential implications for conservation of these species.

## 2. Material and methods

### 2.1 Study area

**Figure 1.**
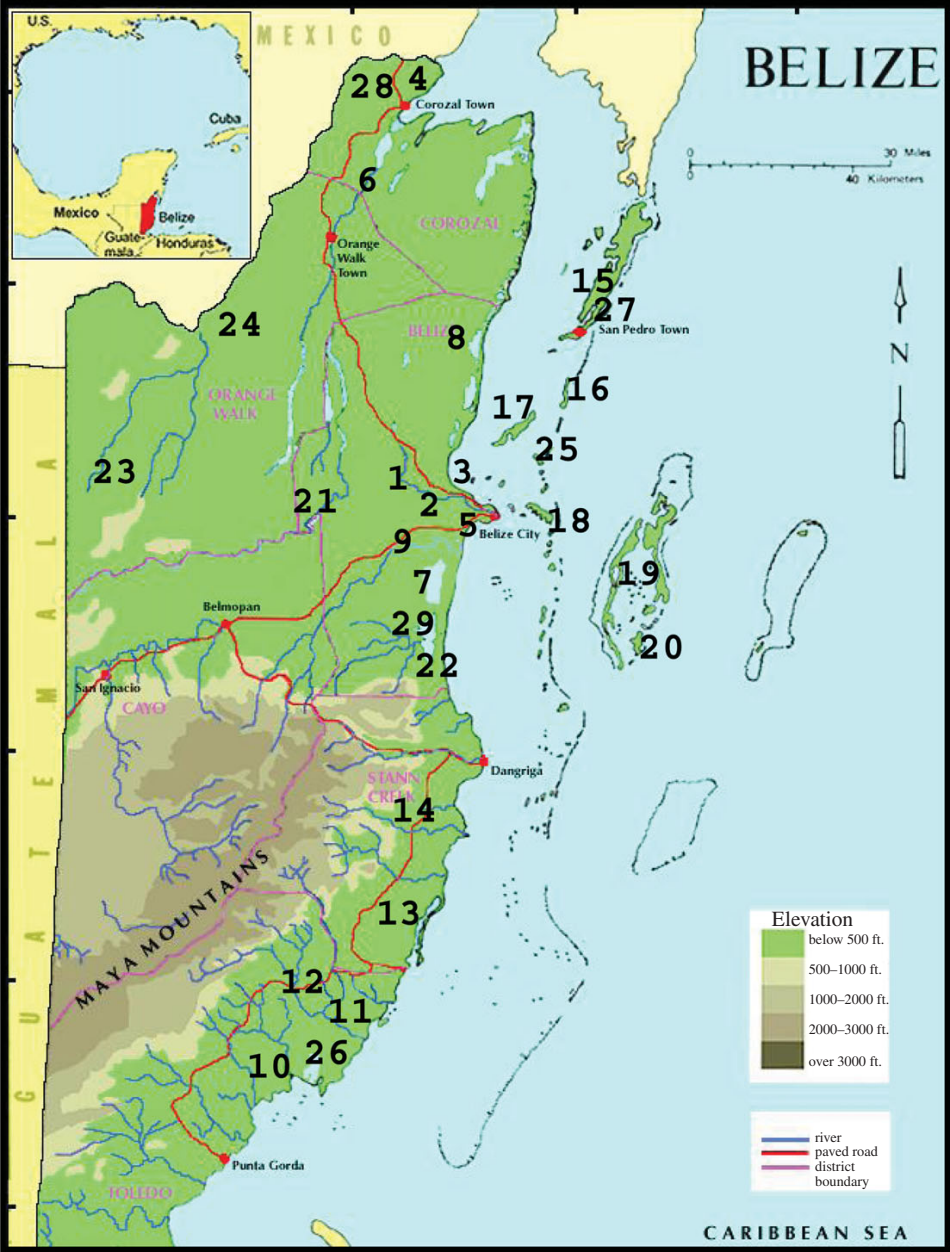
Sampling localities for American (*C. acutus*) and Morelet’s (*C. moreletii*) crocodiles in Belize. Numbers correspond to localities listed in table 1. Adapted from [27].

Our study was conducted in the Caribbean coastal zone of southern Belize (figure 1) [28,29]. The mainland of southern Belize (south of Belize City) is characterized by extensive mangrove swamps and a number of short, swift-flowing rivers (Monkey–Bladen–Swasey River system, and Deep, Moho, Sittee, Temash and Sarstoon rivers) draining the Maya Mountains [29,30]. The Belize barrier reef extends 220 km along the coast, separated from the mainland by the Inner Channel, which contains approximately 450 low elevation islands, or Cays, including three from this study (Turneffe and Lighthouse Atolls, and Glovers Reef). The coastal zone of Belize is described in greater detail elsewhere [28,31,32].

### 2.2 Sampling

Crocodiles of both species (*C. acutus* and *C. moreletii*) were captured as part of a countrywide population survey in the coastal zone from June 1996 through to October 1997 (table 1) [33,34]. Crocodiles were captured at night with the aid of a spotlight; smaller crocodiles (total length [*TL*]≤100 cm) were taken by hand or dip-net, and a noose-pole was used to capture larger individuals (*TL*>100 cm).

**Table 1.** Summary of localities for American crocodile (*C. acutus*) and Morelet’s crocodile (*C. moreletii*) samples collected in coastal mainland habitats of Belize (1996–1997) (adapted from [27]). (Numbers correspond to map in figure 1.)

locality	*C. acutus*	*C. moreletii*
(1) Belize River	0	1
(2) Burdon Canal—FabersLagoon	0	2
(3) Coastline (Ladyville)	0	1
(4) Four-mile Lagoon	0	1
(5) Haulover Creek	0	1
(6) New River	0	15
(7) Northern Lagoon	1	1
(8) Northern River Lagoon	3	0
(9) SibunRiver/BurdonCanal	0	3
(10) Deep River	1	0
(11) Monkey River	0	7
(12) Bladen River	0	7
(13) Placencia Lagoon	0	1
(14) Sittee River	0	4
(15) Ambergris Cay	7	0
(16) Cay Caulker	4	0
(17) Hicks Cay	1	0
(18) Maps Cay	1	0
(19) Turneffe Atoll	22	0
(20) Calabash Cay	2	0
(21) Cox Lagoon	0	1
(22) Gales Point	3	0
(23) Gallon Jug	0	3
(24) Gold Button Lagoon	0	3
(25) Long Cay	2	0
(26) Payne’s Creek	0	3
(27) San Pedro Lagoon	1	0
(28) Sapote Lagoon	0	4
(29) Western Lagoon	3	0
	total *n*=54	56

We recorded standard morphometric measurements from each crocodile, counted the number of dorsal precaudal scale rows and the scales in each row as in Platt *et al*. [35] and noted the presence or absence of irregular subcaudal scale groups [18,36]. Based on published keys, crocodiles exhibiting groups of irregular subcaudal scales and more than four scales in any transverse dorsal precaudal scale row were classified as *C. moreletii*, while those lacking groups of irregular subcaudal scales and having no more than four scales in any dorsal precaudal scale row were classified as *C. acutus* [36]. Crocodiles with atypical characters were classified as possible hybrids.

Approximately 1 ml of blood was drawn from the nuchal sinus of each crocodile [37] and immediately decanted into an equal amount of buffer (10 mM Tris, pH 7.6). Samples were initially stored at room temperature and later at −20°C for long-term storage. All individuals were permanently marked for future identification by notching the dorsal edge of a unique series of caudal scutes [38], and then released at the site of capture within 24 h.

In response to local reports of atypical crocodile nest mounds (described as having mixed features of both *C. acutus* and *C. moreletii*) along rivers in southern Belize [27], we searched this region from March to May during 1997 and 1998. We measured the dimensions of each nest mound and distance to the water (measured from the centre of the mound). We then carefully opened the nest, determined the clutch size and measured (length and width to nearest 0.1 mm) and weighed (±1.0 g) each egg. Egg viability was determined by the presence of opaque bands, and the date of oviposition was estimated by the extent of banding [39]. A single egg was sacrificed from each nest and the embryo preserved in 75% ethanol for later genetic analysis.

### 2.3 Laboratory procedures

Total genomic DNA was extracted from 56 *C. moreletii* and 54 *C. acutus* samples collected from throughout the coastal zone of Belize. Additionally, total genomic DNA was extracted from seven embryos collected at atypical nests found along the Bladen, Swasey and Monkey rivers, and Paynes Creek in southern Belize. For extractions, we used 2 μl of whole blood from adults and a small piece of heart muscle dissected from embryos and isolated DNA using the Qiagen DNeasy Blood and Tissue Kit. Resulting DNA was quantified via gel electrophoresis on a 1% agarose gel and subsequently diluted as necessary to ensure amplification.

We sequenced mtDNA gene regions including 12 s rRNA, 16 s and control region (Dloop) using primers described in Hekkala *et al.* [40]. PCR amplifications were carried out in 25 μl reaction volume containing 1 μl each 10 mM primer and 2 μl template with GE PCR-ready-to-go beads pre-loaded in a 0.2 μl tube to which 21 μl ddH_2_0 was added. Thermocycler parameters for all gene regions consisted of a 5 min denaturation at 94°C for 30 s, 49°C for 30 s and 72°C for 45 s, followed by an extension period of 5 min at 72°C. Bands were visualized on a 1% agarose gel and PCR products were cleaned according to the manufacturer’s instructions using Qiagen PCR cleanup kit. Double-stranded PCR products were sequenced on an ABI 3730XL automated DNA sequencer, and edited and aligned in Sequencher v. 4.5 (Gene Codes, Ann Arbor, MI, USA).

We screened 18 *Crocodylus*-derived dinucleotide microsatellites [14] (table 2) in an 8 μl reaction volume consisting of 1 μl *Taq* polymerase (Perkins Elmer), 0.3 μl of each primer (10 mM), 2.5 μl *Taq* buffer containing 15 μM MgCl_2_, 2.5 μl dNTPs and 0.5 μl of diluted (1:10) template DNA. Amplicons were visualized by electrophoresis in 1.5% agarose and diluted 1:2 with distilled water. A 2 μl aliquot of dilute (1:3) sample was added to a total volume of 8 μl formanide and ROX size standard and run on Applied Biosystems 3100 or 3730 DNA Analyzer. Fragments were analysed using Genemapper^®^ 4.0 (Applied Biosystems).

**Table 2.** Mitochondrial primers [40] and microsatellite primers characterized by Fitzsimmons *et al*. [14] tested for use in identifying hybrids between *C. acutus* and *C. moreletii*. (Fixed marker indicates alleles unique to parental species. Variable marker indicates frequency variation in alleles between parental species.)

	primer name	primer sequence (5′–3′)	species	repeat motif	amplification	fixed	variable
mtDNA	12s	F: CCGTCTTTGACAGTC					
		R: ATGTTCCAAGCACACCTTCC					
	16s	F: AAGGTAGCGTAATCATTTG					
		R: GGGGATTGCGCTGTTATCCCTG					
	Dloop	F: GCCGACATTCTTATTAAACTAC					
		R: GCAGATAAATGAATGCCTTAT					
microsatellite	Cj119	F: GTTTGCTGTGGAATGTTTCTAC	*C. johnsoni*	(CA)14	yes	yes	yes
		R: CGCTATATGAAACGGTGGCTG					
	C391	F: ATGAGTCAGGTGGCAGGTTC	*C. acutus*	(CA)22	yes	no	yes
		R: CATAAATACACTTTTGAGCAGCAG					
	Cj104	F: TCCTTCCATGCATGCACGTGTG	*C. johnsoni*	(CA)12	yes	yes	yes
		R: GTTTCAGTGTCTGGTATTGGAGAAGG					
	Cj105	F: CAACAGAAAGTGCCACCTCAAG	*C. johnsoni*	(CA)14	multiple bands	no	no
		R: GTTTGATTATGAGACACCGCCACC					
	Cj107	F: ACCCCGCATTCTGCCAAGGTG	*C. johnsoni*	(CA)16	multiple bands	no	no
		R: GTTTATTGCCATCCCCACTGTGTC					
	Cj122	F: GTTTCATGCTGACTGTTTCTAATCACC	*C. johnsoni*	(CA)ls	yes	mono	mono
		R: GGAACTACAATTGGTCAACCTCAC					
	Cj127	F: CCCATAGTTTCCTGTTACCTG	*C. johnsoni*	(CT)7TT(CT)12 (CA)16	yes	no	yes
		R: GTTTCCCTCTCTGACTTCAGTGTTG					
	Cj128	F: ATTGGGGCAGATAAGTGGACTC	*C. johnsoni*	(CA)22	no	no	no
		R: GTTTCTGCTTCTCTTCCCTACCTGG					
	Cj35	F: GTTTAGAAGTCTCCAAGCCTCTCAG	*C. johnsoni*	(CT)7TA(CA)17(CT)12	yes	yes	yes
		R: CTGGGGCAAGGATTTAACTCTC					
	Cj101	F: ACAGGAGGAATGTCGCATAATTG	*C. johnsoni*	(CA)12	yes	no	yes
		R: GTTTATACCGTGCCATCCAAGTTAG					
	Cj131	F: GTTTGTCTTCTTCCTCCTGTCCCTC	*C. johnsoni*	(CA)14	yes	yes	yes
		R: AAATGCTGACTCCTACGGATGG					
	Cjl6	F: CATGCAGATTGTTATTCCTGATG	*C. johnsoni*	(CA)20	yes	unknown	unknown
		R: TGTCATGGTGTCAATTAAACTC					
	Cjl8	F: ATCCAAATCCCATGAACCTGAGAG	*C. johnsoni*	unpublished	yes	yes	yes
		R: CCGAGTGCTTACAAGAGGCTGG					
	Cp10	F: GATTAGTTTTACGTGACATGCA	*C. porosus*	(CA)ls	yes	mono	mono
		R: ACATCAAGTCATGGCAGGTGAG					
	CUD68	F: GCTTCAGCAGGGGCTACC	*C. acutus*	(CA)13	only *C. acutus*	plus/minus	no
		R: TGGGGAAACTGCACTTTAGG					
	CUC20	F: GATCTGCAGTGCAAGAAAG	*C. acutus*	unpublished	yes	yes	yes
		R: GGTTTAGCGGTCACAGTAAC					
	CUD78	F GAAGTGAATGCCATCTATCA	*C. acutus*	(CA)15	yes	mono	mono
		R AATTGCATCCCCTTTTG					
	CUI 108	F: ACTGGCCACAGCTGGGGTA	*C. acutus*	(CA)20	multiple bands	no	no
		R: CCAGCAGCGTGGAGAGCTG					

### 2.4 Molecular analytical approaches

We initially identified diagnostic markers (fixed mtDNA haplotypes and private microsatellite alleles) for parental species using samples from populations of each species from outside of the purported hybrid zone. Subsequently, individuals from the purported hybrid zone were examined for specific patterns of admixture in the distribution of haplotypes, private alleles and phenotype. We used Bayesian assignment methods including Newhybrids v. 1.1 [41] and Structure v. 2.3.4 [42] to infer ancestry and to identify putative hybrids. The Structure analysis was implemented with an admixture model with uncorrelated allele frequencies and without including sample location as a prior. We used 20 replicates for each value of *K* (genetic cluster) ranging from *K*=1−7, with 10 000 000 Markov chain Monte Carlo replicates following an initial burn-in of 1 000 000. We chose a threshold for parental species membership in a cluster at 0.0–0.05 or more than 0.95–1.0 and for hybrids between these boundaries.

We used Anderson & Thompson’s [41] Bayesian method of detecting hybrids that more directly attempts to detect hybrid individuals between two parent species as implemented in Newhybrids v. 1.1. This model infers each individual’s genotype frequency class, or hybrid category, thus providing posterior probabilities that reflect the level of certainty that an individual belongs to a given hybrid class (e.g. F_1_, backcross, purebred). Unlike in Structure, here the parameter of interest (*q*) is a discrete variable with up to six genotype frequency classes (i.e. purebred, F_1_, F_2_, backcross). Individuals were assigned to pure *C. acutus*, pure *C. moreletii* and hybrids (F_1_, F_2_ and both F_1_ backcrosses). Results were based on the average of 10 independent runs each with 1 000 000 iterations following a 100 000 step burn-in using Jeffrey’s priors (following preliminary runs indicating similar results with uniform priors). As in Structure, individuals were identified as purebred based on a *q*_*i*_>0.95. To determine the ability of Newhybrids to identify purebred and hybrid individuals, simulated genotypes were created using Hybridlab [43].

Genotypes were selected from pure *C. moreletii* and *C. acutus* individuals identified in the initial Newhybrids analysis (*q*_*i*_>0.95). Alleles were randomly drawn from the pool of parental genotypes to create 100 simulated purebred *C. moreletii* and *C. acutus* individuals. These new parental genotypes were then used to simulate F_1_, F_2_ and backcrossed populations (100 of each hybrid class). These 600 simulated genotypes were then analysed in Newhybrids under the same protocols described above. Power (number of correctly identified individuals for a category over the actual number of individuals of that category) and accuracy (number of correctly identified individuals for a category over the total number of individuals assigned to that category) were calculated for six *T*_*q*_ values (0.95, 0.9, 0.8, 0.7, 0.6, 0.5).

For all downstream environmental and phenotypic analyses reliant upon hybrid identification *a priori*, we used the more conservative estimate of species identification (ID) from Structure results.

### 2.5 Environmental analyses

Once individuals were identified using molecular data and mapped using GPS coordinates, environmental characters associated with parental species and hybrids in Belize were evaluated using distributional modelling (MAXENT). We compared niche models developed for each parental species against models developed for hybrids and evaluated highest ranking environmental variables for each species’ model for statistical differences [44].

The variables used for niche modelling were then used to test whether or not the habitats of parental crocodile species and hybrids differed in their bioclimatic envelopes, as well as the salinity of their habitats. A site’s environmental variables were assigned to only one individual per crocodile species or hybrid per site. The BIOCLIM data [45] used were acquired from the WorldClim database [45] and were formatted for use in R with the raster package using the *raster* and *extract* functions [46,47]. All tests were conducted using R [47], with multivariate statistics relying on the vegan package [48]. The majority of environmental variables were not normally distributed; accordingly, non-parametric tests were used. Differences between the habitats of the species and hybrid categories of crocodile were tested using PERMANOVA with the function *adonis* [48,49]. Individual environmental variables were then compared between crocodile groups with Kruskal–Wallis (KW) tests (function *kruskal.test*), followed by pairwise comparisons (function *pairwise.wilcox.test*). All KW and pairwise comparison *p*-values were adjusted for multiple testing with the false discovery rate (FDR) correction [50].

### 2.6 Phenotypic data

We examined field collected morphological data on head shape and scalation patterns for differences between genetically determined parent species and hybrids. Specifically, we tested the following variables: the head width to length ratio; the snout width at the fifth maxilla to the snout width at the anterior orbit ratio; the presence, reduction or absence of irregular subcaudal scales; and the mean number of scales in the transverse scale rows. As data were largely non-parametric, FDR corrected Kruskal–Wallis and follow up pairwise comparisons were conducted following procedures for environmental analyses. We also compared egg mass from nests containing genetically identified hybrids to parental species egg mass using an ANOVA. Data were tested for violations of normality and homogeneity of variances [51]. Mean values are presented as ±1 s.d. and results were considered significant at *p*≤0.05.

## 3. Results

### 3.1 Genetic characterization of phenotypically identified hybrid crocodiles

Samples from 110 crocodiles were sequenced for three mtDNA gene regions totalling 1374 nucleotides (12 s=365 bp, 16 s=319 bp, control region=690 bp). For analyses of nuclear markers, we were unable to consistently amplify all six loci in 34 individuals resulting in a reduced sample size for nuclear analyses (*n*=76).

As in prior phylogenetic analyses [52], sequences from pure *C. acutus* and *C. moreletii* samples exhibited fixed differences between parental species at multiple sites (12 s=7 bp, 16 s=4 bp, control region=21 bp), which were subsequently considered to be diagnostic characters. Sequenced mtDNA haplotypes revealed 24 individuals field identified as *C. moreletii* or possible hybrids that exhibited *C. acutus* fixed mtDNA markers. The majority of these crocodiles (80%) had been characterized as putative hybrids in the field on the basis of nest type or scale counts.

Of the 18 microsatellite loci tested, 13 produced reliable amplification products in both species and one (Cu68) amplified only in *C. acutus* (table 2). After an initial examination of allelic distribution between *C. moreletii* and *C. acutus*, seven loci (Cj18, Cj131, Cj119, Cj127, Cj35, Cj101 and Cj104) exhibited fixed markers for each parental species and thereafter were characterized for the remainder of the samples.

**Figure 2.**
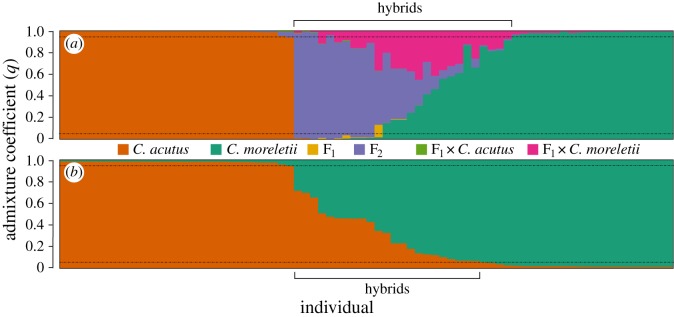
Bayesian assignments of 76 *C. acutus*, *C. moreletii* and hybrid individuals computed by Newhybrids ((*a*) *K*=6, number of genotype frequency classes) and Structure ((*b*) *K*=2, number of species). Each individual is represented by a single vertical line broken into segments whose length is proportional to the estimated membership (probability *q*_*i*_) in the clusters. The ‘hybrid’ identification includes individuals that fall in the 0.05<*q*_*i*_<0.95 range.

The Bayesian clustering analyses performed on simulated genotypes in Newhybrids resulted in clearly identifiable partitions among parental species and hybrids (table 3 and figure 2, upper panel). Power and accuracy were consistently high for both purebred categories across all *T*_*q*_ values, meaning nearly all assigned genotypes were correctly assigned to each parental species (table 4). Accuracy remained near 0.9 across all classes examined for more conservative *T*_*q*_ values (i.e. greater than or equal to 0.8), though power tended to be low for F_2_ and backcrosses. When all hybrid classes were considered together (‘hybrid’), simulations exhibited high levels of power and accuracy in identifying hybrid individuals. Given the number of loci used, our limited ability to correctly assign individuals to F_2_ and backcross populations is expected. Accordingly, we combined posterior probabilities of all hybrid classes as an estimate for the detection of hybrids. We used a conservative threshold of 0.95 to assign individuals as pure or hybrid. At this threshold, our analyses identified 27 hybrids (approx. 35%). These results were similar to those of our Structure analyses (table 3 and figure 2, lower panel). Both Bayesian clustering approaches assigned the majority of parental individuals (97%) to their own species with 99–100% certainty (table 4). Individuals of each parental species were never identified as the other parental species. Only *C. moreletii* (11%) and hybrids (9%) were mis-assigned and then only infrequently (6%). Accuracy of Field ID relative to genetic assignment was highest for *C. acutus* and lower for both *C. moreletii* (82%) and hybrids (92%) (table 5). The accuracy of field ID’d hybrids is probably owing to targeted sampling for those individuals and may not reflect ease of identification in regular surveys.

**Table 3.** Proportions and frequencies of pure and admixed *C. acutus* and *C. moreletii* individuals inferred using Bayesian clustering (Structure) and assignment (Newhybrids) methods. (Only strict assignment (*T*_*q*_=0.95) to parental and admixed classes included.)

	*C. acutus*	admixed	*C. moreletii*
Newhybrids	29 (38.2%)	27 (35.5%)	20 (26.3%)
Structure	29 (38.2%)	23 (30.3%)	24 (31.6%)

**Table 4.** Power and accuracy of Newhybrids to detect pure and hybrid individuals across six *T*_*q*_ values. (All six pure and hybrid classes (excluding ‘hybrid’) consisted of 100 simulated genotypes. The ‘hybrid’ class was created by summing the assignment probabilities of the four hybrid categories and was used to assess the ability of Newhybrids to identify generic ‘hybrid’ individuals. Power is defined as the number of correctly identified individuals for a category over the actual number of individuals of that category and accuracy as the number of correctly identified individuals for a category over the total number of individuals assigned to that category.)

	*T*_*q*_=0.95		*T*_*q*_=0.9		*T*_*q*_=0.8		*T*_*q*_=0.7		*T*_*q*_=0.6		*T*_*q*_=0.5	
class	power	accuracy	power	accuracy	power	accuracy	power	accuracy	power	accuracy	power	accuracy
*C. acutus*	0.96	0.96	0.96	0.96	0.96	0.96	0.97	0.94	0.97	0.94	0.99	0.93
*C. moreletii*	0.93	0.98	0.94	0.98	0.98	0.97	1.00	0.97	1.00	0.97	1.00	0.96
F_1_	0.51	0.98	0.84	0.97	0.96	0.96	0.97	0.94	0.97	0.94	0.98	0.94
F_2_	0.51	1.00	0.55	1.00	0.62	1.00	0.63	0.98	0.65	0.98	0.65	0.98
F_1_×*C. acutus*	0.00	—	0.10	0.91	0.50	0.89	0.70	0.88	0.79	0.89	0.87	0.90
F_1_×*C. moreletii*	0.00	—	0.21	1.00	0.61	0.84	0.75	0.81	0.87	0.82	0.91	0.81
hybrid	0.92	1.00	0.93	1.00	0.94	1.00	0.96	1.00	0.97	1.00	0.97	1.00

**Table 5.** Accuracy of field ID relative to genetic assignment for each category of species ID for pure and admixed *C. acutus* and *C. moreletii* individuals.

field ID	gene ID	% accuracy
*C. acutus*	*C. acutus*	100
*C. moreletii*	*C. moreletii*	82
hybrid	hybrid	92
*C. acutus*	hybrid	n.a.
*C. moreletii*	hybrid	17
hybrid	*C. acutus*	n.a.
hybrid	*C. moreletii*	8

Overall, the results of the Newhybrids assignment and the comparison of diagnostic nuclear alleles with mtDNA haplotypes revealed a consistent pattern of hybridization between female *C. acutus* and male *C. moreletii* indicting unidirectional outcrossing. Five of six embryos from atypical nests and several hybrid adults exhibited combined mtDNA haplotypes and multilocus genotypes consistent with F_2_ backcrossing, and thus hybrid viability.

### 3.2 Phenotypic and reproductive attributes of hybrids

Genetic probability of assignment to parental species versus hybrid was strongly associated with morphological characters relating to head shape and scalation pattern were significant (figure 4): the head width to length ratio (KW *χ*^2^=20.7; FDR corrected, *p*<0.001); the snout width at the fifth maxilla to the snout width at the anterior orbit ratio (KW *χ*^2^=23.1; FDR corrected, *p*<0.001); the presence, reduction or absence of subcaudals (KW *χ*^2^=48.2; FDR corrected, *p*<0.001); and the mean number of scales in the transverse scale rows (KW *χ*^2^=23.8; FDR corrected, *p*<0.001; figure 4). Most significant was the presence, reduction or absence of subcaudal scale rows; presence was fixed for *C. moreletii*and absence was fixed for *C. acutus*, while hybrids tended to be present or reduced (rarely absent). In addition, the mean number of scales in transverse scale rows was the most significant continuous variable. Pairwise comparisons found *C. moreletii*and the hybrids to significantly differ from *C. acutus* for all variables, while hybrids were significantly different for the presence, reduction or absence of the subcaudal scales (*p*=0.005)

Nesting occurred during the dry season and the mean estimated laying date was 22 April ± 8 days (range=10–30 April). During nesting season, 11 atypical crocodile nests were observed along Payne’s Creek (*n*=3), and Monkey (*n*=2), Bladen (*n*=3), Swasey (*n*=2), Sennis (*n*=1) rivers in southern Belize during field surveys in 1997 and 1998. Nests along Monkey and Bladen River were found beside oxbow lakes adjacent to the river, while the remaining nests were constructed on sandbars along the main river channel. All of the nests we examined were mound-type nests, although nest material varied depending on microhabitat. Nests at oxbow lakes were constructed of soil, leaf litter and woody debris, while those along main river channels were composed almost wholly of sand. Mounds (*n*=11) averaged 158±60 cm wide (range=95–300 cm) and 61±24 cm high (range=30–100 cm), and the distance to water ranged from 110 to 1260 cm.

Ten of the 11 nests we examined contained eggs, while one nest had been depredated prior to our arrival. Mean clutch size was 31.3±11.7 eggs (range=15–48 eggs). We measured the linear dimensions of 311 eggs (including three eggs from which the contents had leaked); mean length and width were 77.8±6.5 mm (range=61.3–95.0 mm) and 45.9±3.7 mm (range=36.9–51.1 mm), respectively. Three hundred and eight intact eggs were weighed; mean egg mass was 105.1±20.9 g (*n*=308; range=59–142 g) and 17 (5.5%) were non-viable. Mean clutch size of hybrid containing nests in southern Belize was significantly greater than values reported for either *C. moreletii* or *C. acutus* (table 6; *F*_2,96_=3.97; Tukey–Kramer minimum significant difference; *p*<0.05). Likewise, mean egg mass of hybrid containing nests was significantly greater than reported for either *C. moreletii* or *C. acutus* [53,54] (table 5; paired *t*-test 14.554; *p*<0.01). Seventeen (5.5%) of 308 intact eggs were non-viable.

**Table 6.** (*a*) Crocodile egg mean weights (g) for eggs from typical (*C. acutus* and *C. moreletii*) and atypical crocodile nests. (*b*) ANOVA for all groups indicating significant differences, paired *t*-tests indicated differences between eggs from either parental species and those found in atypical, purported hybrid nests (*p*<0.001).

(*a*) species	*n*	s.d.	range	mean (g)	
*C. acutus*[53]	280	9.7	61.5–111.0	85.6	
*C. moreletii*[21]	1702	9.4	46.2–91.1	69.0	
‘hybrid’ nests	308	20.3	59–142	105.1	

(*b*) source of variation	sum of squares	d.f.	variance	*F*	*p*
between groups	369 990.0	2	184 995.0	1396.0	<0.001
within groups	303 063.1	2287	132.5		
total	673 053.1	2289			

### 3.3 Niche conservatism in parental and hybrid crocodiles

**Figure 3.**
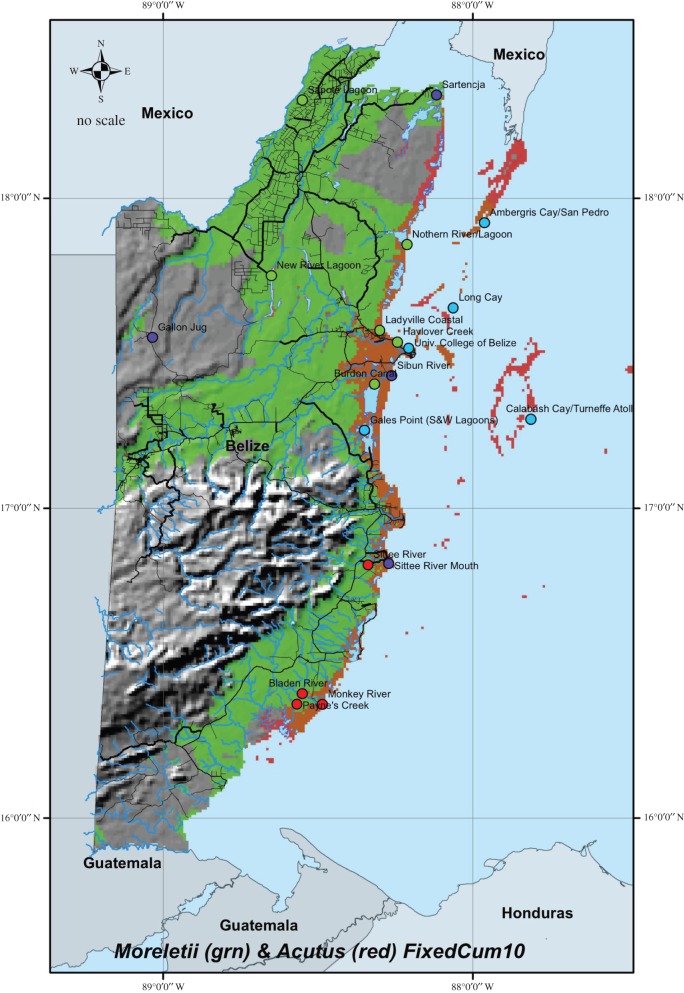
Maximum entropy (MAXENT) species distribution model (SDM) for genetically identified *C. moreletii* (in green) and *C. acutus* (in red) in Belize. Sampling localities for *C. moreletii* (green dots), *C. acutus* (blue dots), hybrids (red dots) and both hybrids and parental (purple dots).

**Figure 4.**
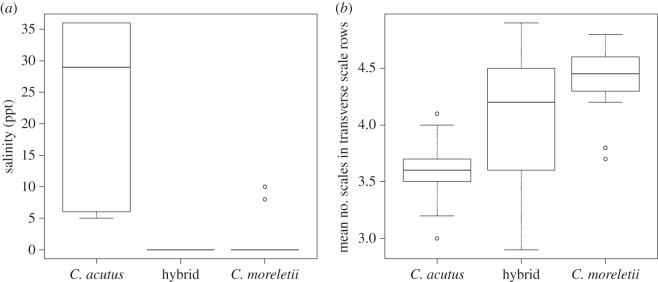
(*a*,*b*) Box-and-whisker plots of the most significant environmental (FDR corrected, *p*=0.012) and continuous morphological variables (FDR corrected, *p*< 0.001) associated with genetically determined species ID for *C. moreletii* and *C. acutus* in Belize using Kruskal–Wallis tests. The box contains the middle two quartiles (separated by the median), the whiskers are the extreme values up to 1.5 times the interquartile range, and the dots represent outliers.

Although distribution mapping of genetically identified *C. acutus* and *C. moreletii* results in narrow areas of overlap (figure 3), overall, species and hybrids did not differ based on combined habitat environmental features (*r*^2^=0.188; *p*=0.111). None of the BIOCLIM environmental variables were significant after correcting for multiple testing. However, BC6 (minimum temperature of coldest month) and BC7 (temperature annual range) were significant before FDR corrections, while BC2 (mean diurnal range) and BC15 (precipitation seasonality) approached significance before correcting. Salinity was the only environmental variable to remain significant after FDR corrections for multiple testing (KW *χ*^2^=14.7; FDR corrected, *p*=0.012; figure 4). Habitat salinity for *C. acutus*was significantly different from that found for *C. moreletii* (*p*=0.010) and for hybrids (*p*=0.003), while the latter two groups did not differ significantly (*p*=0.222).

## 4. Discussion

Analysis of mtDNA from individuals collected outside of areas of sympatry in Belize, as well as data published elsewhere [10,11,40,52], indicates that there are fixed, diagnostic, haplotypic differences between *C. moreletii* and *C. acutus*, which can be used as DNA barcodes. Additionally, nuclear microsatellite loci exhibit both frequency differences and private alleles useful in differentiating between the two species of *Crocodylus* in Belize. These and published species descriptions based on phenotypic characters clearly support their continued recognition as two species.

Our combined use of mtDNA and nuclear markers indicate that hybridization between *C. moreletii* and *C. acutus* has occurred in two regions of Belize: the lower reaches of New River and Rio Bravo around Chetumal Bay, and several coastal rivers in southern Belize, south of Gales Point (figure 3). Similarly, Ray *et al*. [8] detected *C. acutus* haplotypes among crocodiles that phenotypically resembled *C. moreletii* in the New River and Belize River watersheds in northern Belize and concluded hybridization was also occurring in these regions. The presence of discordant species-specific mtDNA haplotypes, multilocus genotypes and phenotypic characteristics confirms the presence of hybrids in these areas. Unlike other studies that found hybrids were cryptic and not readily distinguished on the basis of morphology [8], we found morphologically intermediate characters and atypical nests as relatively reliable indicators of the presence of hybrid crocodiles (table 5 and figure 4); for example, a reduction in subcaudal scales is diagnostic for hybrids when present.

Our use of biparental and maternally inherited markers indicates unidirectional hybridization in southern Belize with male *C. moreletii* crossing with female *C. acutus*. This contrasts with results from Mexico, where Cedeño-Vázquez *et al*. [10] found that hybridization between *C. moreletii* and *C. acutus* is bidirectional, and occurs in about the same proportion in each direction. Asynchrony in courtship and mating in Belize’s crocodile populations may contribute to the observed pattern in our data, where breeding in *C. acutus* occurs during February and March [53], while *C. moreletii* breeds in April and May [54]. We speculate that male *C. moreletii* establish territory during the latter part of *C. acutus* breeding season and breed with female *C. acutus* before female *C. moreletii* enter a reproductive state.

Other factors influencing directionality of geneflow may be behavioural. Unidirectional hybridization is frequent [55] when one species is larger than the other, and males of the larger species usually mate with females of the smaller species [56]. Mating between females of the larger species and males of the smaller species generally does not occur because females rarely select smaller males as mates [56]. Crocodilians engage in elaborate courtship and mating rituals that involve female choice based primarily on the size of male suitors [1]. During courtship and mating, larger males typically dominate breeding groups and drive off or even inflict injuries on smaller subdominant males [1]. Although male *C. acutus* are known to reach maximum TLs of 6–7 m [57], in Belize males rarely attain lengths of over 3 m [33]. By contrast, male *C. moreletii* can reach lengths of 3.6–4.0 m [58,59] and possibly larger [60], suggesting that large male *C. moreletii* would probably displace male *C. acutus* during courtship for access to female *C. acutus*.

Our distribution models indicate that distributions of *C. moreletii* and *C. acutus* are largely related to water salinity (figure 4). Published natural history data suggest that high salinities (approx. 36 ppt) restrict *C. moreletii* to freshwater and mainland coastal habitats [34,61], while *C. acutus* occurs in marine habitats, with lower numbers found in mainland coastal habitats [24,33]. In our study area, populations of *C. moreletii* occur in freshwater wetlands, while *C. acutus* is found primarily on offshore islands and atolls. While the two species occasionally co-occur in brackish mangrove swamps of the coastal mainland [33,34,62–64], our niche models suggest a latitudinal gradient within Belize in niche overlap, with southern populations exhibiting the steepest shift between species (figure 3).

Although our knowledge of the historic distribution remains problematic, a lack of specimen-based records [63] suggest it was absent from this region until recently [64]. An ongoing range expansion by *C. moreletii* into southern Belize may have occurred when populations rebounded rapidly following legal protection in 1981 [34]. Expanding *C. moreletii* populations in Belize are heavily biased in favour of males [34,59,65].

Observed niche conservatism, in combination with known coastal development suggests that hybridization between *C. moreletii* and *C. acutus* in southern Belize may be driven at least partially by recent anthropogenic factors. The ongoing development of coastal and offshore nesting beaches used by *C. acutus* [33,66] might result in the dispersal of female *C. acutus* to less disturbed habitats in southern Belize where contact with male *C. moreletii* would be more likely.

Hybridization in animals is generally regarded as maladaptive because the fitness of hybrid progeny is often reduced [67]. However, in our study, hybrid crocodiles deposited significantly larger clutches than either *C. moreletii* or *C. acutus*, and despite considerable overlap in egg mass, eggs produced by hybrids were significantly larger than eggs of either parental species. Among hybrid *Crocodylus* in captivity, there is no evidence of decreased fitness or dysgenesis; in fact, hybrids produce high-quality skins, grow faster, exhibit enhanced survivorship and produce larger clutches than parental species [3,10]. Because egg size is positively correlated with hatchling size in crocodilians [68–70] and larger hatchlings exhibit accelerated growth and increased survivorship when compared to smaller hatchlings [71], the large eggs deposited by hybrid crocodiles in southern Belize may produce neonates with greater fitness than either *C. moreletii* or *C. acutus* hatchlings. Larger eggs often also contain more water, an advantage for dry season nesting when dehydration can reduce fitness if it interferes with embryonic development near the end of incubation [72,73]. Furthermore, hybridization of *C. moreletii* and *C. acutus* might impart increased salinity tolerance to the offspring, an obvious advantage for crocodiles living in coastal habitats [10]; however, hybrids found in this study exclusively were found in non-saline environments.

The reproductive consequences of hybridization in crocodilians are poorly understood [10]. While genetic data indicate backcrossing in the wild [12], we are unaware of any published reports describing nesting ecology among known hybrid crocodiles in the wild. We found that nest construction and nesting phenology of hybrid crocodiles in southern Belize had elements in common with both *C. moreletii* (mound) and *C. acutus* (sand), while egg and clutch attributes differed from either parental species. In Belize, *C. acutus* generally deposits clutches in shallow holes excavated in the deep sand, while *C. moreletii* constructs mound nests using adjacent vegetation [53,66]. Mound nesting behaviour has occasionally been noted among Florida populations of *C. acutus* [74] and is thought to be an adaptive response to nesting in areas where the probability of flooding is high [57].

Our genetic data for adults and embryos indicate that hybrid crocodiles in southern Belize are fertile and actively reproducing. Contrary to many hypotheses regarding reduced viability in hybrids, our data indicated that only 5% of eggs in hybrid nests were not viable as compared to a range of 8–10% for pure *C. acutus* [33] and 8% for pure *C. morelleti* [54]. Our data appear congruent with Rodriguez *et al*. [11] who concluded that hybrids were not being selected against.

### 4.1 Conservation implications

In Belize, populations of *C. acutus* and *C. moreletii* were nearly extirpated owing to over-harvesting by commercial skin hunters [60,75]. Legal protection was afforded to both species in 1981 and *C. moreletii* populations quickly rebounded [34]. However, recovery of *C. acutus* populations has been slow, largely owing to the continuing destruction of critical nesting habitat [33,66] occurring on the Atlantic and Pacific coasts of Mexico, Central America, as well as the Caribbean Islands of Cuba, Jamaica, Hispaniola and the southern tip of Florida, USA [57]. Despite the more restricted distribution of *C. moreletii* in the Atlantic and Caribbean lowlands of Mexico, Guatemala and Belize [3], recovery has been rapid.

In many instances, natural hybridization is part of the evolutionary process [76]. However, introgression between *C. moreletii* and *C. acutus* in southern Belize is potentially being driven by a combination of anthropogenic factors related to uneven rates of recovery for each species and ongoing destruction of *C. acutus* nesting habitat. Coastal land development and recreational use by tourists occurs predominantly along stretches of raised sandy beach ideal for nesting by *C. acutus*. The accelerated pace of development in coastal Belize over the past two decades may have displaced breeding females to less optimal nesting areas in the south.

Because of asymmetric breeding seasons, such displacement would result in female *C. acutus* in breeding condition encountering male *C. moreletii* prior to the onset of breeding condition in female *C. moreletii* [77]. While introgression of *C. acutus* genomic elements into *C. moreletii* populations appears unrelated to viability, our data suggest that hybridization may act as a sink for *C. acutus* populations if there is differential survival of hybrid offspring [78].

Hybridization is especially problematic when rare species come into contact with other species that are more abundant, and can result in the formation of localized hybrid swarms and eventual genetic swamping of the rarer species [76,79,80]. Given the rarity of *C. acutus* in the region [81], we agree with Cedeño-Vázquez *et al.* [10] that conservation efforts should be focused on this species, but at present management options to prevent further genetic introgression in southern Belize appear limited. Perhaps the best measure is to maintain and attempt to increase *C. acutus* populations on offshore islands and atolls where high salinities preclude the encroachment of *C. moreletii*. Population recruitment of *C. acutus* in Belize is best accomplished through protection of existing nesting beaches and associated nursery habitats that are critical for the survival of hatchlings [33,66] where anthropogenic occupation and alteration of brackish and estuarine waterways may impact important nursery areas for developing young.

Offshore populations of *C. acutus* are expected to remain genetically pure, and expanding populations could act as a source for dispersers destined for the mainland. However, if hybrid progeny exhibit increased salinity tolerance as suggested by Cedeño-Vázquez *et al*. [10], then offshore populations may not function as a genetic refuge for pure *C. acutus* in Belize.

Because of the potential consequences, additional studies to clarify the extent of hybridization and the forces driving hybridization in southern Belize are warranted. New surveys of southern Belize focused on identifying previously marked individuals and hybrid nests are necessary to determine the persistence of hybridization and survival rates of hybrids in the 15 years since surveys and sampling took place. Ultimately, hybridization presents a management problem for New World crocodiles and complicates identification of species based on morphology alone [5]. We concur with Rodriguez *et al*. [11] that future conservation of crocodilians will require genetic identification of pure populations, and ultimately management of those populations, while carefully considering the implications of both natural and anthropogenically reinforced hybridization.

## Data accessibility

The data supporting this article are available at the Dryad digital repository: http://dx.doi.org/10.5061/dryad.32h8t.

## References

[1] Lang JW 1987 Crocodilian behaviour: implications for management. In Wildlife management: crocodiles and alligators (eds GJW Webb, SC Manolis, PJ Whitehead), pp 273–294.Sydney, Australia: Surrey Beatty & Sons.

[2] Honegger R, Hunt R 1990 Breeding crocodiles in zoological gardens outside the species range, with some data on the general situations in European zoos, 1989. In *Crocodiles.**Proc. of the 10th Working Meeting of the IUCN/SSC Crocodile Specialist Group*, Gainesville, FL, USA, 1990, pp. 200–228. Cambridge, UK: IUCN.

[3] Thorbjarnarson JB, Messel H, King FW, Ross JP 1992 Crocodiles: an action plan for their conservation. Cambridge, UK: IUCN.

[4] Fitzsimmons NN, Buchan JC, Lam PV, Polet G, Hung TT, Thang NQ, Gratten J 2002 Identification of purebred *Crocodylus siamensis* for reintroduction in Vietnam. J. Exp. Zool. 294, 373–381. (doi:10.1002/jez.10201) 12461816 10.1002/jez.10201

[5] Weaver JP, Rodriguez D, Venegas-Anaya M, Cedeño-Vázquez JR, Forstner MR, Densmore LD 2008 Genetic characterization of captive Cuban crocodiles (*Crocodylus rhombifer*) and evidence of hybridization with the American crocodile (*Crocodylus acutus*). J. Exp. Zool. A Ecol. Genet. Physiol. 309, 649–660. (doi:10.1002/jez.471) 18646197 10.1002/jez.471

[6] Varona LS 1987 The status of *Crocodylus acutus* in Cuba. Caribbean J. Sci. 23, 256–259.

[7] Ramos R, de Buffrenil V, Ross J 1994 Current status of the Cuban crocodile, *Crocodylus rhombifer*, in the wild. In *Crocodiles, Proc. of the 12th Working Meeting of the Crocodile Specialist Group*, 1994, pp. 113–140. Darwin, Australia: Crocodile Specialist Group.

[8] Ray DA et al. 2004 Low levels of nucleotide diversity in *Crocodylus moreletii* and evidence of hybridization with *C. acutus*. Conserv. Genet. 5, 449–462. (doi:10.1023/B:COGE.0000041024.96928.fe)

[9] Russello M, Brazaitis P, Gratten J, Watkins-Colwell G, Caccone A 2007 Molecular assessment of the genetic integrity, distinctiveness and phylogeographic context of the saltwater crocodile (*Crocodylus porosus*) on Palau. Conserv. Genet. 8, 777–787. (doi:10.1007/s10592-006-9225-7)

[10] Cedeño-Vázquez JR, Rodriguez D, Calme S, Ross JP, Densmore LD, Thorbjarnarson JB 2008 Hybridization between *Crocodylus acutus* and *Crocodylus moreletii* in the Yucatan Peninsula: I. Evidence from mitochondrial DNA and morphology. J. Exp. Zool. A Ecol. Genet. Physiol. 309, 661–673. (doi:10.1002/jez.473) 18626922 10.1002/jez.473

[11] Rodriguez D, Cedeño-Vázquez JR, Forstner MR, Densmore LD 2008 Hybridization between *Crocodylus acutus* and *Crocodylus moreletii* in the Yucatan Peninsula: II. Evidence from microsatellites. J. Exp. Zool. A Ecol. Genet. Physiol. 309, 674–686. (doi:10.1002/jez.499) 18800373 10.1002/jez.499

[12] Milián-García Y, Ramos-Targarona R, Pérez-Fleitas E, Sosa-Rodríguez G, Guerra-Manchena L, Alonso-Tabet M, Espinosa-López G, Russello M 2014 Genetic evidence of hybridization between the critically endangered Cuban crocodile and the American crocodile: implications for population history and *in situ*/*ex situ* conservation. Heredity 114, 272–280. (doi:10.1038/hdy.2014.96) 25335559 10.1038/hdy.2014.96 PMC4815585

[13] Ross JP, Espinosa E 1998 Crocodiles: status survey and conservation action plan. Gland, Switzerland: IUCN.

[14] FitzSimmons N, Tanksley S, Forstner M, Louis E, Daglish R, Gratten J, Davis S 2001 Microsatellite markers for *Crocodylus*: new genetic tools for population genetics, mating system studies and forensics. In Crocodilian biology and evolution (eds GC Grigg, F Seebacher, CE Franklin), pp. 51–57. Chipping Norton, UK: Surrey Beatty and Sons.

[15] Hekkala E 2004 Conservation genetics at the species boundary: case studies from African and Caribbean crocodiles (Genus: Crocodylus). New York, NY: Columbia University.

[16] Schmidt KP 1924 Notes on Central American crocodiles. Chicago, IL: University of Illinois.

[17] Powell J 1972 The Morelet’s crocodile: an unknown quantity. Smith III. Animal Kingdom.

[18] Ross CA, Ross FD 1974 Caudal scalation of central American *Crocodylus*. Proc. Biol. Soc. Wash. 87, 231–234.

[19] Abercrombie CL, Davidson D, Hope CA, Scott DE 1980 Status of Morelet’s crocodile *Crocodylus moreleti* in Belize. Biol. Conserv. 17, 103–113. (doi:10.1016/0006-3207(80)90040-3)

[20] Ross FD, Mayer GC 1983 On the dorsal armor of the Crocodilia. Smith VII. In Advances in herpetology and evolutionary biology (eds A Rhodin, K Miyata), pp. 305–331. Cambridge, MA: Museum of Comparative Zoology.

[21] Platt SG 1996 The ecology and status of Morelet’s crocodile in Belize. PhD dissertation, Clemson University, Clemson, SC, USA.

[22] Sigler L 1998 A *Crocodylus acutus* with the appearance of a *C. moreletii*. Crocodile Spec. Group Newslett. 173, 9–11.

[23] Villegas A 2005 Phenotypic characteristics of *Crocodylus acutus* and *C. moreletii* in South Quintana Roo. Crocodile Spec. Group Newslett. 24,8–9.

[24] Cedeño-Vázquez JR, Ross JP, Calmé S 2006 Population status and distribution of *Crocodylus acutus* and *C. moreletii* in southeastern Quintana Roo, Mexico. Herpetol. Nat. History 10, 17–30.

[25] Nature IU. f. C. o., Resources N. 2008 IUCN red list of threatened species. Cambridge, UK: International Union for Conservation of Nature and Natural Resources.

[26] Ross JP, Espinosa E 1998 Crocodiles: status survey and conservation action plan, 2nd edn. Gland, Switzerland: IUCN/SSC Crocodile Specialist Group.

[27] Platt S, Thorbjarnarson J 1997 Status and life history of the American crocodile in Belize. *Belize coastal zone management project BZE/92 G*. 31.

[28] Platt SG, Meerman JC, Rainwater TR 1999 Diversity, observations and conservation of the herpetofauna of Turneffe, Lighthouse, and Glovers Atolls, Belize. Br. Herpetol. Soc. Bull. 66, 1–13.

[29] McField M, Wells S, Gibson J 1996 State of the coastal zone report, Belize, 1995. Coastal Zone Management Programme. Belize City: Belize: CZMAI.

[30] Hartshorn G et al. 1984 Belize country profile: a field study. Belize City, Belize: USAID and Robert Nicolait and Assoc., Ltd.

[31] Stoddart DR 1962 Three Caribbean atolls: Turneffe Islands, Lighthouse Reef, and Glover’s Reef. British Honduras: DTIC Document.

[32] Zisman S, Planning F 1992 Mangroves in Belize: their characteristics, use and conservation.Forest Planning and Management Project. Belmopan, Belize: Belize Forest Department.

[33] Platt SG, Thorbjarnarson JB 2000 Status and conservation of the American crocodile, *Crocodylus acutus*, in Belize. Biol. Conserv. 96, 13–20. (doi:10.1016/S0006-3207(00)00038-0)

[34] Platt SG, Thorbjarnarson JB 2000 Population status and conservation of Morelet’s crocodile, *Crocodylus moreletii*, in northern Belize. Biol. Conserv. 96, 21–29. (doi:10.1016/S0006-3207(00)00039-2)

[35] Platt S 2008 Scalation of Morelet’s crocodile (*Crocodylus moreletii*) from Northern Belize. Herpetol. Rev. 39, 293.

[36] Platt S, Rainwater T 2005 A review of morphological characters useful for distinguishing Morelet’s crocodile (*Crocodylus moreletii*) and American crocodile (*Crocodylus acutus*) with an emphasis on populations in the coastal zone of Belize. Bull. Chicago Herpetol. Soc. 40, 25–29.

[37] Olson G, Hessler J, Faith R 1975 Technics for blood collection and intravascular infusion of reptiles. Lab. Anim. Sci. 25, 783–786. 1207049

[38] Jennings ML, David DN, Portier KM 1991 Effect of marking techniques on growth and survivorship of hatchling alligators. Wildl. Soc. Bull. 19, 204–207.

[39] Ferguson MW 1985 Reproductive biology and embryology of the crocodilians. Biol. Reptilia 14, 329–491.

[40] Hekkala E et al. 2011 An ancient icon reveals new mysteries: mummy DNA resurrects a cryptic species within the Nile crocodile. Mol. Ecol. 20, 4199–4215. (doi:10.1111/j.1365-294X.2011.05245.x) 21906195 10.1111/j.1365-294X.2011.05245.x

[41] Anderson E, Thompson E 2002 A model-based method for identifying species hybrids using multilocus genetic data. Genetics 160, 1217– 1229. 11901135 10.1093/genetics/160.3.1217 PMC1462008

[42] Pritchard JK, Stephens M, Donnelly P 2000 Inference of population structure using multilocus genotype data. Genetics 155, 945–959. 10835412 10.1093/genetics/155.2.945 PMC1461096

[43] Nielsen EE, Bach LA, Kotlicki P 2006 HYBRIDLAB (version 1.0): a program for generating simulated hybrids from population samples.Mol. Ecol. Notes 6, 971–973. (doi:10.1111/j.1471-8286.2006.01433.x)

[44] Pearson RG, Raxworthy CJ 2009 The evolution of local endemism in Madagascar: watershed versus climatic gradient hypotheses evaluated by null biogeographic models. Evolution 63, 959–967. (doi:10.1111/j.1558-5646.2008.00596.x) 19210532 10.1111/j.1558-5646.2008.00596.x

[45] Hijmans RJ, Cameron SE, Parra JL, Jones PG, Jarvis A 2005 Very high resolution interpolated climate surfaces for global land areas. Int. J. Climatol. 25, 1965–1978. (doi:10.1002/joc.1276)

[46] Hijmans RJ, van Etten J 2012 Raster: geographic data analysis and modeling. R package v. 2.1–49.See https://cran.r-project.org/web/packages/raster/index.html.Illinois.

[47] R Development Core Team. 2014 R: a language and environment for statistical computing. Vienna, Austria: R Foundation for Statistical Computing.

[48] Oksanen J 2013 Vegan: ecological diversity. See hokudai. ac. jp (accessed 15 August 2014).

[49] Anderson MJ 2001 A new method for non-parametric multivariate analysis of variance. Austral. Ecol. 26, 32–46.

[50] Benjamini Y, Hochberg Y 1995 Controlling the false discovery rate: a practical and powerful approach to multiple testing. J. R. Stat. Soc. B 57, 289–300.

[51] Zar JH 1999 Biostatistical analysis. The University of Michigan, MI: Pearson Education India.

[52] Meredith RW, Hekkala ER, Amato G, Gatesy J 2011 A phylogenetic hypothesis for *Crocodylus* (Crocodylia) based on mitochondrial DNA: evidence for a trans-Atlantic voyage from Africa to the New World. Mol. Phylogenet. Evol. 60, 183–191. (doi:10.1016/j.ympev.2011.03.026) 21459152 10.1016/j.ympev.2011.03.026

[53] Platt SG, Thorbjarnarson JB, Price A 2000 Nesting ecology of the American crocodile in the coastal zone of Belize. Copeia. 2000,869–873. (doi:10.1643/0045-8511%282000%29000%5B0869%3ANEOTAC%5D2.0.CO%3B2)

[54] Platt SG, Rainwater TR, Thorbjarnarson JB, McMurry ST 2008 Reproductive dynamics of a tropical freshwater crocodilian: Morelet’s crocodile in northern Belize. J. Zool. 275, 177–189. (doi:10.1111/j.1469-7998.2008.00426.x)

[55] Wirtz P 1999 Mother species–father species: unidirectional hybridization in animals with female choice. Anim. Behav. 58, 1–12. (doi:10.1006/anbe.1999.1144) 10413535 10.1006/anbe.1999.1144

[56] Grant PR, Grant BR 1997 Mating patterns of Darwin’s finch hybrids determined by song and morphology. Biol. J. Linn. Soc. 60, 317–343. (doi:10.1111/j.1095-8312.1997.tb01499.x)

[57] Thorbjarnarson JB 1989 Ecology of the American crocodile, Crocodylus acutus. In Crocodiles, their ecology, management and conservation, a special publication of the Crocodile Specialist Group, pp. 228–259, Gland, Switzerland: IUCN Publications.

[58] Perez-Higareda G, Rangel-Rangel A, Smith H 1991 Maximum sizes of Morelet’s and American crocodiles. Bull. MD Herpetol. Soc. 27, 34–37.

[59] Platt SG, Rainwater TR, Thorbjarnarson JB, Finger AG, Anderson TA, McMurry ST 2009 Size estimation, morphometrics, sex ratio, sexual size dimorphism, and biomass of Morelet’s crocodile in northern Belize. Caribb. J. Sci. 45, 80–93.

[60] Frost MD 1974 A biogeographical analysis of some relationships between man, land, and wildlife in Belize (British Honduras). Corvallis, OR: Oregan State University.

[61] Escobedo-Galvan A, Palacios-Chavez V, Vovides-Tejera A 2008 *Crocodylus moreletii* (Morelet’s Crocodile) salinity tolerance. Herpetol. Rev. 39, 346–347.

[62] Meerman J 1992 The status of crocodiles in the eastern Corozal District. Occas. Papers Belize Nat. Hist. Soc. 1, 1–5.

[63] Lee JC 1996 The amphibians and reptiles of the Yucatan Peninsula. Ithaca, NY: Comstock.

[64] Platt SG, Thorbjarnarson JB, Rainwater TR 1999 Distribution of Morelet’s crocodile (*Crocodylus moreletii*) in southern Belize. Southwestern Nat. 44, 395–398.

[65] Rainwater T, Platt S, McMurry S 1998 A population study of Morelet’s crocodile (*Crocodylus moreletii*) in the New River watershed of northern Belize. In *Proc. of the 14th Working Meeting of the Crocodile Specialist Group, 1998*, pp. 206–220. Darwin, Australia: Crocodile Specialist Group.

[66] Platt SG, Rainwater TR, Nichols S 2004 A recent population assessment of the American crocodile (Crocodylus acutus) in Turneffe Atoll, Belize. Herpetolog. Bull. 89, 26–32.

[67] Arnold ML 1997 Natural hybridization and evolution: Oxford series in ecology and evolution. Oxford, UK: Oxford University Press.

[68] Staton MA, Dixon JR 1977 Breeding biology of the spectacled caiman, Caiman crocodilus crocodilus, in the Venezuelan Llanos. Darwin, Australia: Crocodile Specialist Group, Department of the Interior, US Fish and Wildlife Service.

[69] Deitz DC, Hines TC 1980 Alligator nesting in north-central Florida. Copeia 1980, 249–258. (doi:10.2307/1444001)

[70] Campos Z, Magnusson W 1995 Relationships between rainfall, nesting habitat and fecundity of *Caiman crocodilus yacare* in the Pantanal, Brazil. J. Trop. Ecol. 11, 351–358. (doi:10.1017/S0266467400008828)

[71] Messel H, Vorlicek GC 1989 Ecology of Crocodylus porosus in northern Australia. In Crocodiles: their ecology, management and conservation, pp. 164–183. Gland, Switzerland: IUCN.

[72] Packard GC, Packard MJ, Miller K, Boardman TJ 1987 Influence of moisture, temperature, and substrate on snapping turtle eggs and embryos. Ecology 68, 983–993. (doi:10.2307/1938369)

[73] Campos Z, Magnusson W, Sanaiotti T, Coutinho M 2008 Reproductive trade-offs in *Caiman crocodilus crocodilus* and *Caiman crocodilus yacare*: implications for size-related management quotas. Herpetol. J. 18, 91–96.

[74] Kushlan JA, Mazzotti FJ 1989 Population biology of the American crocodile. J. Herpetol. 23, 7–21. (doi:10.2307/1564310)

[75] Charnock-Wilson J 1970 Manatees and crocodiles. Oryx 10, 236–238. (doi:10.1017/S0030605300008498)

[76] Allendorf FW, Leary RF, Spruell P, Wenburg JK 2001 The problems with hybrids: setting conservation guidelines. Trends Ecol. Evol. 16, 613–622. (doi:10.1016/S0169-5347(01)02290-X)

[77] Aguilar X, Casas-Andreu G 1991 Crocodylus acutus (American crocodile) reproduction. Life history notes Herp. Rev. 22, 98.

[78] Ralls K, Harvey PH, Lyles AM 1986 Inbreeding in natural populations of birds and mammals. In *Conservation biology: the science of scarcity and diversity* (ed. ME Soule), pp. 35–56. Sunderland, MA: Sinauer Associates.

[79] Rhymer JM, Simberloff D 1996 Extinction by hybridization and introgression. Annu. Rev. Ecol. Syst. 27, 83–109. (doi:10.1146/annurev.ecolsys.27.1.83)

[80] Olden JD, Poff NL, Douglas MR, Douglas ME, Fausch KD 2004 Ecological and evolutionary consequences of biotic homogenization. Trends Ecol. Evol. 19, 18–24. (doi:10.1016/j.tree.2003.09.010) 16701221 10.1016/j.tree.2003.09.010

[81] Thorbjarnarson J et al. 2006 Regional habitat conservation priorities for the American crocodile. Biol. Conserv. 128, 25–36. (doi:10.1016/j.biocon.2005.09.013)

